# 3-[5-Methyl-1-(4-methyl­phen­yl)-1*H*-1,2,3-triazol-4-yl]-*N*-phenyl-5-[4-(piperidin-1-yl)phen­yl]-4,5-dihydro-1*H*-pyrazole-1-carbothio­amide dimethyl­formamide hemisolvate

**DOI:** 10.1107/S1600536812024488

**Published:** 2012-06-02

**Authors:** Bakr F. Abdel-Wahab, Hanan A. Mohamed, Seik Weng Ng, Edward R. T. Tiekink

**Affiliations:** a Applied Organic Chemistry Department, National Research Centre, Dokki, 12622 Giza, Egypt; b Department of Chemistry, University of Malaya, 50603 Kuala Lumpur, Malaysia; c Chemistry Department, Faculty of Science, King Abdulaziz University, PO Box 80203 Jeddah, Saudi Arabia

## Abstract

The essentially planar pyrazole ring (r.m.s. deviation = 0.013 Å) in the title hemisolvate, C_31_H_33_N_7_S·0.5C_3_H_7_NO, is almost coplanar with the pendant thio­urea residue [N—N—C—S torsion angle = −173.2 (4)°] and slightly twisted with respect to the triazole ring [dihedral angle = 7.7 (3)°]. An intra­molecular thio­urea–pyrazole N—H⋯N hydrogen bond, *via* an *S*(5) loop, is formed. Supra­molecular chains along the *c* axis are formed in the crystal *via* piperidine–triazole C—H⋯N inter­actions. These are bridged into loosely associated double chains *via* C—H⋯O inter­actions involving the disordered (over two positions) dimethyl­formamide solvent mol­ecules. The thio­urea-bound phenyl ring is also disordered over two positions of equal occupancy.

## Related literature

For the biological activity of related compounds, see: Abdel-Wahab *et al.* (2009 ▶, 2012*a* ▶). For a related pyrazolyl-1,2,3-triazole structure, see: Abdel-Wahab *et al.* (2012*b* ▶).
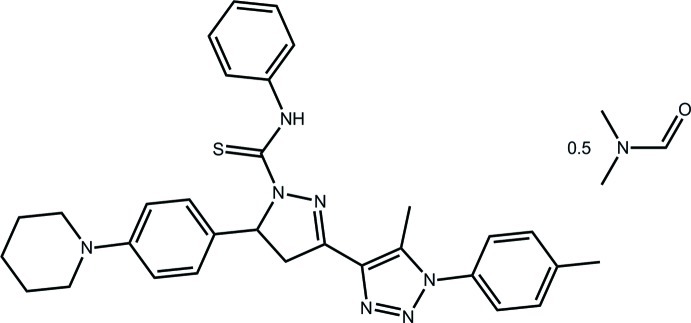


## Experimental

### 

#### Crystal data

C_31_H_33_N_7_S·0.5C_3_H_7_NO
*M* *_r_* = 522.26
Monoclinic, 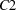

*a* = 42.077 (4) Å
*b* = 5.9274 (5) Å
*c* = 12.0737 (11) Å
β = 105.665 (9)°
*V* = 2899.5 (4) Å^3^
*Z* = 4
Cu *K*α radiation
μ = 1.29 mm^−1^
*T* = 100 K
0.35 × 0.15 × 0.05 mm

#### Data collection

Agilent SuperNova Dual diffractometer with an Atlas detector
Absorption correction: multi-scan (*CrysAlis PRO*; Agilent, 2012 ▶) *T*_min_ = 0.661, *T*_max_ = 0.938
10437 measured reflections
3317 independent reflections
3229 reflections with *I* > 2σ(*I*)
*R*_int_ = 0.037

#### Refinement

*R*[*F* ^2^ > 2σ(*F* ^2^)] = 0.091
*wR*(*F* ^2^) = 0.214
*S* = 1.11
3317 reflections
396 parameters
41 restraints
H-atom parameters constrained
Δρ_max_ = 0.71 e Å^−3^
Δρ_min_ = −1.06 e Å^−3^

### 

Data collection: *CrysAlis PRO* (Agilent, 2012 ▶); cell refinement: *CrysAlis PRO*; data reduction: *CrysAlis PRO*; program(s) used to solve structure: *SHELXS97* (Sheldrick, 2008 ▶); program(s) used to refine structure: *SHELXL97* (Sheldrick, 2008 ▶); molecular graphics: *ORTEP-3* (Farrugia, 1997 ▶) and *DIAMOND* (Brandenburg, 2006 ▶); software used to prepare material for publication: *publCIF* (Westrip, 2010 ▶).

## Supplementary Material

Crystal structure: contains datablock(s) global, I. DOI: 10.1107/S1600536812024488/xu5554sup1.cif

Structure factors: contains datablock(s) I. DOI: 10.1107/S1600536812024488/xu5554Isup2.hkl

Supplementary material file. DOI: 10.1107/S1600536812024488/xu5554Isup3.cml

Additional supplementary materials:  crystallographic information; 3D view; checkCIF report

## Figures and Tables

**Table 1:** Hydrogen-bond geometry (Å, °)

*D*—H⋯*A*	*D*—H	H⋯*A*	*D*⋯*A*	*D*—H⋯*A*
N1—H1⋯N3	0.88	2.13	2.597 (8)	112
C13—H13*A*⋯O1	0.98	2.56	3.466 (14)	154
C28—H28*B*⋯N5^i^	0.99	2.57	3.355 (10)	137

Symmetry code: (i) 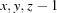.

**Fig. 1.**
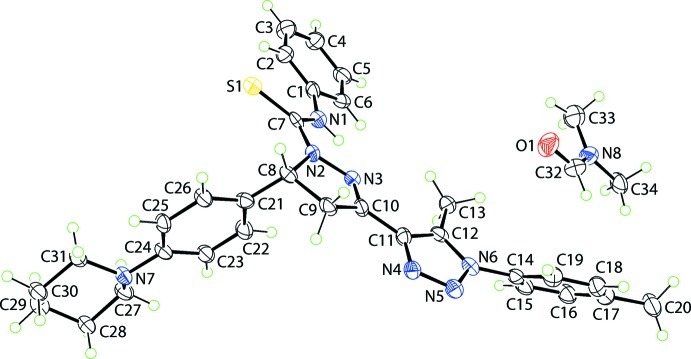
The molecular structure of (I) showing the atom-labelling scheme and displacement ellipsoids at the 50% probability level.

**Fig. 2.**
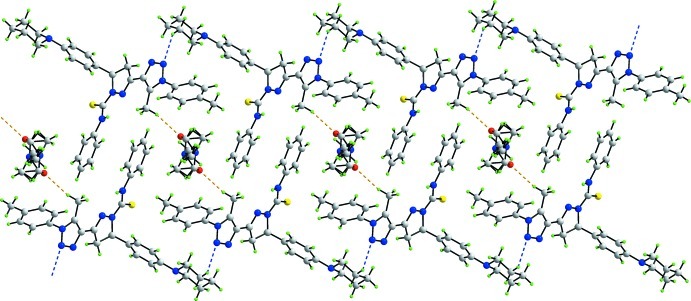
A view of a supramolecular chain along the c axis in (I). The dimethylformamide molecule is disordered over two positions. The C—H···O and C—H···N interactions are shown as orange and blue dashed lines, respectively.

## supplementary crystallographic information

### Comment

The synthesis and crystallographic characterization of the title compound was motivated by the established biological activities exhibited by related 3-(benzofuran-2-yl)-4,5-dihydro-5-phenyl-1-(4-phenylthiazol-2-yl)-1*H*-pyrazole and 1,2,3-triazol-4-yl-pyrazolylthiazoles (Abdel-Wahab *et al.* 2012*a*; Abdel-Wahab *et al.* 2009) and accompanying structural studies (Abdel-Wahab *et al.*, 2012*b*).

In (I), Fig. 1, the pyrazole ring is planar with a r.m.s. deviation = 0.013 Å; the maximum deviation is 0.005 (6) Å for the N2 atom. The thiourea group is close to co-planar with the ring [the N3—N2—C7—S1 torsion angle = -173.2 (4)°] and the connected triazole ring is slightly twisted out of the plane [dihedral angle = 7.7 (3)°]. There is a significant twist between the triazole and attached benzene rings with the dihedral angle being 49.4 (3)°. The conformation of the piperidinyl ring is close to a chair. The thiourea-N—H atom is orientated towards the pyrazole-N3 atom and forms a hydrogen bond *via* an *S*(5) loop, Table 1.

In the crystal packing, connections between molecules are of the type piperidinyl-C—H···N(triazole), Table 1, leading to the formation of a supramolecular chain along the *c* axis. Two chains are connected by the (disordered) dimethylformamide molecules *via* methyl-C13—H···O1 interactions into a loosely associated double chain, Fig. 2 and Table 1.

### Experimental

The title compound was prepared according to the reported method (Abdel-Wahab *et al.*, 2012*a*). Crystals were obtained from its DMF solution by slow evaporation at room temperature.

### Refinement

The H-atoms were placed in calculated positions [N—H = 0.88 Å; C—H = 0.95 to 0.98 Å, *U*_iso_(H) = 1.2–1.5*U*_eq_(*N*,*C*)] and were included in the refinement in the riding model approximation.

The phenyl ring is disordered over two positions in an assumed 1:1 ratio; the ring was refined as a rigid hexagon with 1.39 Å sides. The displacement factors of the primed atoms were set to those of the unprimed ones.

The DMF molecule is disordered over a twofold rotation axis. The C—O distance was restrained to 1.25±0.01 Å and the *N*–C_carbonyl_ distance to 1.35±0.01 Å; the other two N–C distances were restrained to 1.45±0.01 Å. Additionally, the 1,3-related distances were also restrained. The anisotropic displacement parameters were tightly restrained to be nearly isotropic.

The absolute structure could not be refined despite the presence of a sulfur atom as the crystal is a racemic twin; 1153 Friedel pairs were merged.

The maximum and minimum residual electron density peaks of 0.71 and 1.06 e Å^-3^, respectively, were located 0.71 Å and 0.82 Å, respectively, from the S1 atom.

A reflection, *i.e*. (-3 1 3), was omitted owing to poor agreement.

### Crystal data

**Table:**

C_31_H_33_N_7_S·0.5C_3_H_7_NO	*F*(000) = 1216
*M**_r_* = 522.26	*D*_x_ = 1.311 Mg m^−^^3^
Monoclinic, *C*2	Cu *K*α radiation, λ = 1.54184 Å
Hall symbol: C 2y	Cell parameters from 6358 reflections
*a* = 42.077 (4) Å	θ = 3.8–76.9°
*b* = 5.9274 (5) Å	µ = 1.29 mm^−^^1^
*c* = 12.0737 (11) Å	*T* = 100 K
β = 105.665 (9)°	Prism, light-brown
*V* = 2899.5 (4) Å^3^	0.35 × 0.15 × 0.05 mm
*Z* = 4	

### Data collection

**Table:**

Agilent SuperNova Dual diffractometer with an Atlas detector	3317 independent reflections
Radiation source: SuperNova (Cu) X-ray Source	3229 reflections with *I* > 2σ(*I*)
Mirror monochromator	*R*_int_ = 0.037
Detector resolution: 10.4041 pixels mm^-1^	θ_max_ = 77.1°, θ_min_ = 3.8°
ω scan	*h* = −52→49
Absorption correction: multi-scan (*CrysAlis PRO*; Agilent, 2012)	*k* = −5→7
*T*_min_ = 0.661, *T*_max_ = 0.938	*l* = −14→15
10437 measured reflections	

### Refinement

**Table:**

Refinement on *F*^2^	Secondary atom site location: difference Fourier map
Least-squares matrix: full	Hydrogen site location: inferred from neighbouring sites
*R*[*F*^2^ > 2σ(*F*^2^)] = 0.091	H-atom parameters constrained
*wR*(*F*^2^) = 0.214	*w* = 1/[σ^2^(*F*_o_^2^) + (0.0422*P*)^2^ + 29.4613*P*] where *P* = (*F*_o_^2^ + 2*F*_c_^2^)/3
*S* = 1.11	(Δ/σ)_max_ = 0.001
3317 reflections	Δρ_max_ = 0.71 e Å^−^^3^
396 parameters	Δρ_min_ = −1.06 e Å^−^^3^
41 restraints	Extinction correction: *SHELXL97* (Sheldrick, 2008), Fc^*^=kFc[1+0.001xFc^2^λ^3^/sin(2θ)]^-1/4^
Primary atom site location: structure-invariant direct methods	Extinction coefficient: 0.00150 (17)

### Special details

**Table:**

Geometry. All e.s.d.'s (except the e.s.d. in the dihedral angle between two l.s. planes) are estimated using the full covariance matrix. The cell e.s.d.'s are taken into account individually in the estimation of e.s.d.'s in distances, angles and torsion angles; correlations between e.s.d.'s in cell parameters are only used when they are defined by crystal symmetry. An approximate (isotropic) treatment of cell e.s.d.'s is used for estimating e.s.d.'s involving l.s. planes.

### Fractional atomic coordinates and isotropic or equivalent isotropic displacement parameters (Å^2^)

**Table:**

	*x*	*y*	*z*	*U*_iso_*/*U*_eq_	Occ. (<1)
S1	0.59659 (4)	0.0014 (4)	0.47105 (16)	0.0329 (5)	
N1	0.56945 (14)	0.4011 (13)	0.5037 (5)	0.0306 (14)	
H1	0.5726	0.5285	0.5426	0.037*	
N2	0.61985 (13)	0.3203 (11)	0.6223 (5)	0.0250 (13)	
N3	0.61644 (13)	0.5210 (12)	0.6819 (5)	0.0251 (12)	
N4	0.67600 (14)	0.7524 (12)	0.9292 (5)	0.0282 (13)	
N5	0.67224 (14)	0.9126 (13)	1.0004 (5)	0.0304 (14)	
N6	0.64025 (13)	0.9852 (13)	0.9628 (5)	0.0267 (13)	
N7	0.72517 (13)	0.4072 (12)	0.3176 (5)	0.0261 (13)	
C1	0.53887 (17)	0.3838 (17)	0.4206 (10)	0.0259 (16)	0.50
C2	0.5261 (2)	0.2040 (15)	0.3484 (10)	0.029 (2)	0.50
H2	0.5391	0.0727	0.3489	0.035*	0.50
C3	0.4944 (2)	0.2165 (17)	0.2753 (9)	0.036 (3)	0.50
H3	0.4856	0.0936	0.2259	0.043*	0.50
C4	0.47537 (19)	0.409 (2)	0.2745 (10)	0.032 (3)	0.50
H4	0.4537	0.4172	0.2245	0.038*	0.50
C5	0.4881 (2)	0.5885 (16)	0.3467 (10)	0.034 (3)	0.50
H5	0.4752	0.7199	0.3462	0.041*	0.50
C6	0.5199 (2)	0.5761 (14)	0.4198 (9)	0.029 (2)	0.50
H6	0.5286	0.6989	0.4692	0.035*	0.50
C1'	0.53743 (17)	0.3832 (17)	0.4284 (9)	0.0259 (16)	0.50
C2'	0.5190 (2)	0.1876 (15)	0.4256 (9)	0.029 (2)	0.50
H2'	0.5279	0.0650	0.4749	0.035*	0.50
C3'	0.4875 (2)	0.1716 (15)	0.3507 (10)	0.036 (3)	0.50
H3'	0.4749	0.0380	0.3489	0.043*	0.50
C4'	0.4745 (2)	0.3511 (19)	0.2787 (10)	0.032 (3)	0.50
H4'	0.4530	0.3401	0.2275	0.038*	0.50
C5'	0.4929 (2)	0.5466 (16)	0.2814 (9)	0.034 (3)	0.50
H5'	0.4840	0.6693	0.2322	0.041*	0.50
C6'	0.5244 (2)	0.5627 (14)	0.3563 (10)	0.029 (2)	0.50
H6'	0.5370	0.6963	0.3582	0.035*	0.50
C7	0.59446 (16)	0.2551 (14)	0.5330 (6)	0.0261 (15)	
C8	0.65420 (15)	0.2319 (14)	0.6552 (6)	0.0252 (15)	
H8	0.6543	0.0662	0.6708	0.030*	
C9	0.66858 (15)	0.3640 (15)	0.7684 (6)	0.0281 (16)	
H9A	0.6707	0.2663	0.8366	0.034*	
H9B	0.6904	0.4294	0.7710	0.034*	
C10	0.64330 (16)	0.5443 (13)	0.7621 (5)	0.0240 (15)	
C11	0.64669 (17)	0.7263 (13)	0.8469 (6)	0.0246 (14)	
C12	0.62375 (16)	0.8719 (14)	0.8666 (6)	0.0263 (15)	
C13	0.58794 (16)	0.9065 (15)	0.8053 (6)	0.0305 (16)	
H13A	0.5758	0.9465	0.8612	0.046*	
H13B	0.5788	0.7670	0.7656	0.046*	
H13C	0.5858	1.0285	0.7490	0.046*	
C14	0.62887 (16)	1.1423 (14)	1.0318 (6)	0.0276 (16)	
C15	0.61095 (18)	1.3329 (15)	0.9829 (7)	0.0327 (17)	
H15	0.6065	1.3611	0.9027	0.039*	
C16	0.59990 (19)	1.4774 (16)	1.0516 (7)	0.0354 (17)	
H16	0.5872	1.6041	1.0173	0.043*	
C17	0.6065 (2)	1.4476 (13)	1.1719 (8)	0.0363 (19)	
C18	0.6245 (2)	1.2523 (16)	1.2175 (7)	0.0360 (18)	
H18	0.6292	1.2239	1.2977	0.043*	
C19	0.63557 (19)	1.1015 (16)	1.1493 (6)	0.0343 (17)	
H19	0.6476	0.9712	1.1822	0.041*	
C20	0.5944 (2)	1.6067 (17)	1.2460 (8)	0.047 (2)	
H20A	0.5827	1.5227	1.2929	0.071*	
H20B	0.5793	1.7156	1.1977	0.071*	
H20C	0.6131	1.6871	1.2964	0.071*	
C21	0.67202 (16)	0.2810 (14)	0.5649 (6)	0.0269 (16)	
C22	0.67004 (19)	0.4876 (16)	0.5091 (7)	0.0350 (17)	
H22	0.6565	0.6026	0.5273	0.042*	
C23	0.68695 (17)	0.5317 (14)	0.4285 (6)	0.0305 (16)	
H23	0.6848	0.6751	0.3921	0.037*	
C24	0.70737 (16)	0.3670 (14)	0.3994 (6)	0.0258 (15)	
C25	0.70995 (18)	0.1619 (14)	0.4574 (6)	0.0291 (16)	
H25	0.7240	0.0480	0.4417	0.035*	
C26	0.69243 (17)	0.1197 (13)	0.5380 (6)	0.0280 (15)	
H26	0.6946	−0.0229	0.5751	0.034*	
C27	0.71379 (18)	0.5951 (15)	0.2389 (7)	0.0318 (17)	
H27A	0.7099	0.7279	0.2832	0.038*	
H27B	0.6925	0.5540	0.1838	0.038*	
C28	0.73850 (18)	0.6574 (14)	0.1727 (6)	0.0293 (16)	
H28A	0.7592	0.7105	0.2270	0.035*	
H28B	0.7295	0.7818	0.1187	0.035*	
C29	0.74577 (18)	0.4548 (15)	0.1060 (6)	0.0333 (19)	
H29A	0.7254	0.4055	0.0486	0.040*	
H29B	0.7624	0.4954	0.0650	0.040*	
C30	0.75863 (18)	0.2688 (15)	0.1902 (6)	0.0307 (17)	
H30A	0.7801	0.3149	0.2423	0.037*	
H30B	0.7624	0.1328	0.1479	0.037*	
C31	0.73474 (18)	0.2102 (14)	0.2620 (7)	0.0313 (17)	
H31A	0.7147	0.1397	0.2115	0.038*	
H31B	0.7453	0.0985	0.3215	0.038*	
O1	0.5327 (3)	0.8817 (18)	0.9722 (10)	0.051 (3)	0.50
N8	0.4987 (3)	1.1815 (15)	0.9647 (8)	0.039 (4)	0.50
C32	0.5278 (3)	1.0766 (18)	1.0017 (9)	0.041 (4)	0.50
H32	0.5455	1.1547	1.0528	0.049*	0.50
C33	0.4703 (3)	1.078 (3)	0.8870 (13)	0.051 (5)	0.50
H33A	0.4768	0.9346	0.8593	0.077*	0.50
H33B	0.4615	1.1789	0.8215	0.077*	0.50
H33C	0.4534	1.0509	0.9274	0.077*	0.50
C34	0.4944 (4)	1.4106 (18)	1.002 (2)	0.043 (5)	0.50
H34A	0.5155	1.4673	1.0499	0.064*	0.50
H34B	0.4780	1.4107	1.0460	0.064*	0.50
H34C	0.4867	1.5079	0.9341	0.064*	0.50

### Atomic displacement parameters (Å^2^)

**Table:**

	*U*^11^	*U*^22^	*U*^33^	*U*^12^	*U*^13^	*U*^23^
S1	0.0282 (8)	0.0365 (10)	0.0366 (9)	−0.0056 (9)	0.0133 (7)	−0.0043 (9)
N1	0.025 (3)	0.035 (4)	0.035 (3)	−0.002 (3)	0.013 (2)	−0.005 (3)
N2	0.020 (3)	0.028 (3)	0.029 (3)	0.000 (3)	0.012 (2)	−0.006 (3)
N3	0.024 (3)	0.026 (3)	0.029 (3)	−0.003 (3)	0.012 (2)	−0.002 (3)
N4	0.026 (3)	0.027 (3)	0.035 (3)	0.002 (3)	0.013 (2)	−0.004 (3)
N5	0.024 (3)	0.033 (4)	0.036 (3)	0.001 (3)	0.012 (2)	−0.004 (3)
N6	0.023 (3)	0.031 (3)	0.029 (3)	0.001 (3)	0.012 (2)	0.004 (3)
N7	0.025 (3)	0.026 (3)	0.031 (3)	0.003 (3)	0.013 (2)	0.004 (3)
C1	0.023 (3)	0.028 (4)	0.030 (4)	0.005 (3)	0.014 (3)	0.000 (3)
C2	0.029 (5)	0.025 (5)	0.032 (5)	0.000 (4)	0.009 (4)	−0.001 (5)
C3	0.030 (6)	0.033 (6)	0.043 (6)	0.003 (5)	0.006 (5)	−0.002 (6)
C4	0.026 (4)	0.031 (8)	0.036 (4)	0.001 (5)	0.005 (3)	−0.002 (5)
C5	0.030 (5)	0.030 (6)	0.047 (7)	0.000 (5)	0.019 (5)	0.003 (6)
C6	0.032 (5)	0.022 (5)	0.039 (7)	−0.006 (4)	0.017 (5)	−0.001 (5)
C1'	0.023 (3)	0.028 (4)	0.030 (4)	0.005 (3)	0.014 (3)	0.000 (3)
C2'	0.029 (5)	0.025 (5)	0.032 (5)	0.000 (4)	0.009 (4)	−0.001 (5)
C3'	0.030 (6)	0.033 (6)	0.043 (6)	0.003 (5)	0.006 (5)	−0.002 (6)
C4'	0.026 (4)	0.031 (8)	0.036 (4)	0.001 (5)	0.005 (3)	−0.002 (5)
C5'	0.030 (5)	0.030 (6)	0.047 (7)	0.000 (5)	0.019 (5)	0.003 (6)
C6'	0.032 (5)	0.022 (5)	0.039 (7)	−0.006 (4)	0.017 (5)	−0.001 (5)
C7	0.024 (3)	0.032 (4)	0.030 (3)	−0.003 (3)	0.019 (3)	0.000 (3)
C8	0.016 (3)	0.031 (4)	0.030 (3)	0.001 (3)	0.010 (3)	0.002 (3)
C9	0.016 (3)	0.040 (5)	0.030 (3)	−0.001 (3)	0.008 (2)	−0.001 (3)
C10	0.023 (3)	0.029 (4)	0.024 (3)	0.000 (3)	0.012 (2)	−0.001 (3)
C11	0.026 (3)	0.024 (4)	0.027 (3)	0.004 (3)	0.011 (3)	0.001 (3)
C12	0.022 (3)	0.034 (4)	0.024 (3)	−0.006 (3)	0.009 (3)	0.006 (3)
C13	0.024 (3)	0.033 (4)	0.038 (4)	−0.001 (3)	0.014 (3)	−0.006 (4)
C14	0.021 (3)	0.027 (4)	0.039 (4)	−0.003 (3)	0.014 (3)	−0.005 (3)
C15	0.028 (4)	0.031 (4)	0.044 (4)	0.004 (3)	0.019 (3)	0.004 (4)
C16	0.037 (4)	0.028 (4)	0.048 (4)	0.001 (4)	0.023 (3)	0.002 (4)
C17	0.048 (4)	0.014 (4)	0.057 (5)	−0.002 (3)	0.032 (4)	−0.009 (3)
C18	0.045 (4)	0.034 (4)	0.035 (4)	−0.005 (4)	0.020 (3)	−0.002 (4)
C19	0.037 (4)	0.034 (4)	0.034 (4)	0.001 (4)	0.012 (3)	−0.001 (4)
C20	0.065 (6)	0.034 (5)	0.058 (5)	0.000 (5)	0.044 (5)	−0.001 (5)
C21	0.020 (3)	0.029 (4)	0.032 (4)	−0.003 (3)	0.007 (3)	0.000 (3)
C22	0.036 (4)	0.026 (4)	0.050 (4)	0.003 (4)	0.025 (3)	−0.002 (4)
C23	0.031 (3)	0.025 (4)	0.041 (4)	0.000 (3)	0.019 (3)	0.001 (3)
C24	0.019 (3)	0.027 (4)	0.033 (3)	0.000 (3)	0.012 (3)	0.004 (3)
C25	0.029 (4)	0.024 (4)	0.040 (4)	0.004 (3)	0.019 (3)	−0.002 (3)
C26	0.029 (3)	0.021 (4)	0.040 (4)	0.004 (3)	0.018 (3)	0.005 (3)
C27	0.031 (4)	0.030 (4)	0.038 (4)	−0.001 (3)	0.017 (3)	0.006 (4)
C28	0.026 (3)	0.032 (4)	0.033 (4)	0.002 (3)	0.014 (3)	0.005 (3)
C29	0.029 (3)	0.041 (5)	0.032 (3)	0.003 (3)	0.011 (3)	0.005 (4)
C30	0.027 (3)	0.032 (4)	0.037 (4)	0.001 (3)	0.015 (3)	0.004 (4)
C31	0.030 (4)	0.027 (4)	0.043 (4)	−0.001 (3)	0.020 (3)	0.000 (3)
O1	0.046 (6)	0.048 (7)	0.059 (6)	0.003 (6)	0.017 (5)	−0.019 (6)
N8	0.038 (6)	0.033 (6)	0.050 (7)	−0.003 (6)	0.017 (6)	−0.012 (5)
C32	0.038 (7)	0.041 (8)	0.043 (7)	−0.012 (6)	0.010 (6)	−0.009 (6)
C33	0.048 (8)	0.052 (9)	0.056 (8)	0.000 (7)	0.019 (7)	−0.006 (7)
C34	0.045 (11)	0.039 (7)	0.055 (7)	0.005 (6)	0.032 (7)	−0.003 (8)

### Geometric parameters (Å, º)

**Table:**

S1—C7	1.693 (8)	C13—H13C	0.9800
N1—C7	1.334 (10)	C14—C19	1.391 (10)
N1—C1	1.405 (8)	C14—C15	1.398 (11)
N1—C1'	1.411 (8)	C15—C16	1.359 (11)
N1—H1	0.8800	C15—H15	0.9500
N2—C7	1.354 (9)	C16—C17	1.414 (11)
N2—N3	1.417 (8)	C16—H16	0.9500
N2—C8	1.487 (8)	C17—C18	1.412 (12)
N3—C10	1.282 (8)	C17—C20	1.482 (11)
N4—N5	1.318 (9)	C18—C19	1.378 (11)
N4—C11	1.368 (9)	C18—H18	0.9500
N5—N6	1.369 (8)	C19—H19	0.9500
N6—C12	1.359 (9)	C20—H20A	0.9800
N6—C14	1.416 (10)	C20—H20B	0.9800
N7—C24	1.411 (8)	C20—H20C	0.9800
N7—C31	1.458 (10)	C21—C26	1.382 (10)
N7—C27	1.458 (10)	C21—C22	1.389 (12)
C1—C2	1.3900	C22—C23	1.378 (10)
C1—C6	1.3900	C22—H22	0.9500
C2—C3	1.3900	C23—C24	1.406 (10)
C2—H2	0.9500	C23—H23	0.9500
C3—C4	1.3900	C24—C25	1.393 (11)
C3—H3	0.9500	C25—C26	1.392 (9)
C4—C5	1.3900	C25—H25	0.9500
C4—H4	0.9500	C26—H26	0.9500
C5—C6	1.3900	C27—C28	1.518 (9)
C5—H5	0.9500	C27—H27A	0.9900
C6—H6	0.9500	C27—H27B	0.9900
C1'—C2'	1.3900	C28—C29	1.522 (11)
C1'—C6'	1.3900	C28—H28A	0.9900
C2'—C3'	1.3900	C28—H28B	0.9900
C2'—H2'	0.9500	C29—C30	1.498 (11)
C3'—C4'	1.3900	C29—H29A	0.9900
C3'—H3'	0.9500	C29—H29B	0.9900
C4'—C5'	1.3900	C30—C31	1.534 (9)
C4'—H4'	0.9500	C30—H30A	0.9900
C5'—C6'	1.3900	C30—H30B	0.9900
C5'—H5'	0.9500	C31—H31A	0.9900
C6'—H6'	0.9500	C31—H31B	0.9900
C8—C21	1.509 (9)	O1—C32	1.242 (9)
C8—C9	1.550 (10)	N8—C32	1.338 (8)
C8—H8	1.0000	N8—C33	1.442 (9)
C9—C10	1.495 (10)	N8—C34	1.456 (9)
C9—H9A	0.9900	C32—H32	0.9500
C9—H9B	0.9900	C33—H33A	0.9800
C10—C11	1.467 (10)	C33—H33B	0.9800
C11—C12	1.363 (10)	C33—H33C	0.9800
C12—C13	1.503 (9)	C34—H34A	0.9800
C13—H13A	0.9800	C34—H34B	0.9800
C13—H13B	0.9800	C34—H34C	0.9800
			
C7—N1—C1	130.3 (8)	C12—C13—H13C	109.5
C7—N1—C1'	132.0 (8)	H13A—C13—H13C	109.5
C1—N1—C1'	5.1 (9)	H13B—C13—H13C	109.5
C7—N1—H1	114.9	C19—C14—C15	120.6 (7)
C1—N1—H1	114.9	C19—C14—N6	118.5 (7)
C1'—N1—H1	112.9	C15—C14—N6	120.8 (7)
C7—N2—N3	118.5 (6)	C16—C15—C14	119.1 (7)
C7—N2—C8	127.8 (6)	C16—C15—H15	120.5
N3—N2—C8	112.6 (5)	C14—C15—H15	120.5
C10—N3—N2	106.5 (6)	C15—C16—C17	123.0 (8)
N5—N4—C11	108.2 (6)	C15—C16—H16	118.5
N4—N5—N6	106.9 (6)	C17—C16—H16	118.5
C12—N6—N5	111.0 (6)	C18—C17—C16	116.0 (7)
C12—N6—C14	130.6 (6)	C18—C17—C20	121.5 (8)
N5—N6—C14	118.1 (6)	C16—C17—C20	122.5 (8)
C24—N7—C31	116.9 (6)	C19—C18—C17	122.1 (7)
C24—N7—C27	116.2 (6)	C19—C18—H18	119.0
C31—N7—C27	113.2 (6)	C17—C18—H18	119.0
C2—C1—C6	120.0	C18—C19—C14	119.2 (8)
C2—C1—N1	128.9 (7)	C18—C19—H19	120.4
C6—C1—N1	111.0 (7)	C14—C19—H19	120.4
C1—C2—C3	120.0	C17—C20—H20A	109.5
C1—C2—H2	120.0	C17—C20—H20B	109.5
C3—C2—H2	120.0	H20A—C20—H20B	109.5
C4—C3—C2	120.0	C17—C20—H20C	109.5
C4—C3—H3	120.0	H20A—C20—H20C	109.5
C2—C3—H3	120.0	H20B—C20—H20C	109.5
C5—C4—C3	120.0	C26—C21—C22	117.1 (6)
C5—C4—H4	120.0	C26—C21—C8	119.9 (7)
C3—C4—H4	120.0	C22—C21—C8	122.9 (7)
C4—C5—C6	120.0	C23—C22—C21	122.3 (8)
C4—C5—H5	120.0	C23—C22—H22	118.8
C6—C5—H5	120.0	C21—C22—H22	118.8
C5—C6—C1	120.0	C22—C23—C24	120.8 (8)
C5—C6—H6	120.0	C22—C23—H23	119.6
C1—C6—H6	120.0	C24—C23—H23	119.6
C2'—C1'—C6'	120.0	C25—C24—C23	116.7 (6)
C2'—C1'—N1	120.5 (7)	C25—C24—N7	121.0 (6)
C6'—C1'—N1	119.5 (7)	C23—C24—N7	122.2 (7)
C3'—C2'—C1'	120.0	C24—C25—C26	121.6 (7)
C3'—C2'—H2'	120.0	C24—C25—H25	119.2
C1'—C2'—H2'	120.0	C26—C25—H25	119.2
C4'—C3'—C2'	120.0	C21—C26—C25	121.3 (7)
C4'—C3'—H3'	120.0	C21—C26—H26	119.3
C2'—C3'—H3'	120.0	C25—C26—H26	119.3
C5'—C4'—C3'	120.0	N7—C27—C28	112.2 (6)
C5'—C4'—H4'	120.0	N7—C27—H27A	109.2
C3'—C4'—H4'	120.0	C28—C27—H27A	109.2
C4'—C5'—C6'	120.0	N7—C27—H27B	109.2
C4'—C5'—H5'	120.0	C28—C27—H27B	109.2
C6'—C5'—H5'	120.0	H27A—C27—H27B	107.9
C5'—C6'—C1'	120.0	C27—C28—C29	110.5 (7)
C5'—C6'—H6'	120.0	C27—C28—H28A	109.5
C1'—C6'—H6'	120.0	C29—C28—H28A	109.5
N2—C7—N1	115.0 (7)	C27—C28—H28B	109.5
N2—C7—S1	118.8 (6)	C29—C28—H28B	109.5
N1—C7—S1	126.2 (6)	H28A—C28—H28B	108.1
N2—C8—C21	112.1 (6)	C30—C29—C28	108.0 (6)
N2—C8—C9	100.2 (5)	C30—C29—H29A	110.1
C21—C8—C9	112.9 (6)	C28—C29—H29A	110.1
N2—C8—H8	110.4	C30—C29—H29B	110.1
C21—C8—H8	110.4	C28—C29—H29B	110.1
C9—C8—H8	110.4	H29A—C29—H29B	108.4
C10—C9—C8	102.3 (5)	C29—C30—C31	112.2 (6)
C10—C9—H9A	111.3	C29—C30—H30A	109.2
C8—C9—H9A	111.3	C31—C30—H30A	109.2
C10—C9—H9B	111.3	C29—C30—H30B	109.2
C8—C9—H9B	111.3	C31—C30—H30B	109.2
H9A—C9—H9B	109.2	H30A—C30—H30B	107.9
N3—C10—C11	120.4 (6)	N7—C31—C30	112.6 (7)
N3—C10—C9	115.6 (6)	N7—C31—H31A	109.1
C11—C10—C9	123.8 (6)	C30—C31—H31A	109.1
C12—C11—N4	110.1 (6)	N7—C31—H31B	109.1
C12—C11—C10	130.3 (6)	C30—C31—H31B	109.1
N4—C11—C10	119.3 (6)	H31A—C31—H31B	107.8
N6—C12—C11	103.9 (6)	C32—N8—C33	122.9 (9)
N6—C12—C13	124.6 (7)	C32—N8—C34	120.7 (10)
C11—C12—C13	131.6 (7)	C33—N8—C34	116.4 (12)
C12—C13—H13A	109.5	O1—C32—N8	123.0 (10)
C12—C13—H13B	109.5	O1—C32—H32	118.5
H13A—C13—H13B	109.5	N8—C32—H32	118.5
			
C7—N2—N3—C10	179.4 (6)	C9—C10—C11—N4	8.2 (10)
C8—N2—N3—C10	−11.2 (7)	N5—N6—C12—C11	0.3 (8)
C11—N4—N5—N6	0.6 (8)	C14—N6—C12—C11	−173.0 (8)
N4—N5—N6—C12	−0.6 (8)	N5—N6—C12—C13	178.3 (7)
N4—N5—N6—C14	173.7 (6)	C14—N6—C12—C13	5.0 (12)
C7—N1—C1—C2	2.2 (15)	N4—C11—C12—N6	0.1 (8)
C1'—N1—C1—C2	−110 (7)	C10—C11—C12—N6	173.6 (7)
C7—N1—C1—C6	179.2 (7)	N4—C11—C12—C13	−177.7 (7)
C1'—N1—C1—C6	67 (7)	C10—C11—C12—C13	−4.2 (13)
C6—C1—C2—C3	0.0	C12—N6—C14—C19	125.9 (8)
N1—C1—C2—C3	176.8 (13)	N5—N6—C14—C19	−47.1 (10)
C1—C2—C3—C4	0.0	C12—N6—C14—C15	−53.4 (11)
C2—C3—C4—C5	0.0	N5—N6—C14—C15	133.7 (7)
C3—C4—C5—C6	0.0	C19—C14—C15—C16	−0.5 (11)
C4—C5—C6—C1	0.0	N6—C14—C15—C16	178.7 (7)
C2—C1—C6—C5	0.0	C14—C15—C16—C17	1.8 (12)
N1—C1—C6—C5	−177.4 (11)	C15—C16—C17—C18	−2.1 (12)
C7—N1—C1'—C2'	41.5 (13)	C15—C16—C17—C20	179.8 (8)
C1—N1—C1'—C2'	114 (7)	C16—C17—C18—C19	1.1 (12)
C7—N1—C1'—C6'	−138.2 (8)	C20—C17—C18—C19	179.2 (8)
C1—N1—C1'—C6'	−66 (7)	C17—C18—C19—C14	0.1 (12)
C6'—C1'—C2'—C3'	0.0	C15—C14—C19—C18	−0.5 (11)
N1—C1'—C2'—C3'	−179.8 (11)	N6—C14—C19—C18	−179.7 (7)
C1'—C2'—C3'—C4'	0.0	N2—C8—C21—C26	−139.9 (7)
C2'—C3'—C4'—C5'	0.0	C9—C8—C21—C26	107.8 (8)
C3'—C4'—C5'—C6'	0.0	N2—C8—C21—C22	42.8 (10)
C4'—C5'—C6'—C1'	0.0	C9—C8—C21—C22	−69.5 (9)
C2'—C1'—C6'—C5'	0.0	C26—C21—C22—C23	1.3 (12)
N1—C1'—C6'—C5'	179.8 (11)	C8—C21—C22—C23	178.7 (7)
N3—N2—C7—N1	5.7 (9)	C21—C22—C23—C24	−0.4 (12)
C8—N2—C7—N1	−161.8 (6)	C22—C23—C24—C25	−1.2 (11)
N3—N2—C7—S1	−173.2 (4)	C22—C23—C24—N7	−179.6 (7)
C8—N2—C7—S1	19.2 (9)	C31—N7—C24—C25	24.7 (10)
C1—N1—C7—N2	−176.3 (9)	C27—N7—C24—C25	162.7 (7)
C1'—N1—C7—N2	−169.9 (9)	C31—N7—C24—C23	−156.9 (7)
C1—N1—C7—S1	2.6 (12)	C27—N7—C24—C23	−18.9 (10)
C1'—N1—C7—S1	8.9 (12)	C23—C24—C25—C26	1.8 (11)
C7—N2—C8—C21	64.7 (10)	N7—C24—C25—C26	−179.7 (7)
N3—N2—C8—C21	−103.4 (7)	C22—C21—C26—C25	−0.7 (11)
C7—N2—C8—C9	−175.3 (7)	C8—C21—C26—C25	−178.2 (7)
N3—N2—C8—C9	16.5 (7)	C24—C25—C26—C21	−0.9 (12)
N2—C8—C9—C10	−14.7 (7)	C24—N7—C27—C28	167.4 (6)
C21—C8—C9—C10	104.7 (7)	C31—N7—C27—C28	−53.1 (8)
N2—N3—C10—C11	−174.5 (6)	N7—C27—C28—C29	57.4 (8)
N2—N3—C10—C9	0.0 (8)	C27—C28—C29—C30	−58.0 (8)
C8—C9—C10—N3	10.2 (8)	C28—C29—C30—C31	55.9 (9)
C8—C9—C10—C11	−175.6 (6)	C24—N7—C31—C30	−170.6 (6)
N5—N4—C11—C12	−0.5 (8)	C27—N7—C31—C30	50.2 (8)
N5—N4—C11—C10	−174.8 (6)	C29—C30—C31—N7	−52.8 (9)
N3—C10—C11—C12	9.2 (11)	C33—N8—C32—O1	−0.1 (4)
C9—C10—C11—C12	−164.8 (7)	C34—N8—C32—O1	−179.8 (3)
N3—C10—C11—N4	−177.8 (7)		

### Hydrogen-bond geometry (Å, º)

**Table:**

*D*—H···*A*	*D*—H	H···*A*	*D*···*A*	*D*—H···*A*
N1—H1···N3	0.88	2.13	2.597 (8)	112
C13—H13*A*···O1	0.98	2.56	3.466 (14)	154
C28—H28*B*···N5^i^	0.99	2.57	3.355 (10)	137

Symmetry code: (i) *x*, *y*, *z*−1.
